# Supplementary material for: Diagnostic Strategies and Algorithms for Investigating Cancer Predisposition Syndromes in Children Presenting with Malignancy
Source: Cancers (Basel). 2022 Jul 31;14(15):3741. doi: 10.3390/cancers14153741 (PMC9367486; doi:10.3390/cancers14153741)
Supplement: Supplementary file 1 [file cancers-14-03741-s001.zip › cancers-1794492-supplementary.pdf]

Supplementary Materials: Diagnostic Strategies and Algorithms for Investigating Cancer Predisposition Syndromes in Children Presenting with Malignancy

Linda Rossini, Caterina Durante, Silvia Bresolin, Enrico Opocher, Antonio Marzollo and Alessandra Biffi

Table S1. List of malignancies requiring direct genetic referral following CCSC, JMC and MI-POGG

CCSC	JONGMANS	MIPOGG
Adrenocortical carcinoma	Adrenocortical carcinoma / adenoma	**SNC and ocular tumors**
Aggressive fibromatosis	ALL (low hypodiploid)	Atypical teratoid rhabdoid tumor
Atypical theratoid/rhabdoid tumor	ALL (ring chromosome 21)	Choroid plexus carcinoma
Choroid plexus carcinoma	ALL (Robertsonian translocation 15;21)	Dysplastic cerebellar gangliocytoma
Endolymphatic sac tumour	ALL relapse (*TP53* mutated)	Endolymphatic sac tumor
Gangliocytoma	AML (Monosomy 7)	Hemangioblastoma
Hemangioblastoma	Basal cell carcinoma	Optic pathway glioma
Hepatoblastoma	Botryoid rhabdomyosarcoma of the urogenital tract (fusion-negative)	Pineoblastoma
Hepatocellular carcinoma	Chondromesenchymal harmatoma	Pituitary adenoma
JMML	Choroid plexus carcinoma / tumor	Retinoblastoma
Medulloblastoma (not if known non-SHH subtype and the child is > 3 yo)	Colorectal carcinoma	Subependymal giant cell astrocytoma
Meningioma	Cystic nephroma	Vestibular schwannoma
MDS	Endolymphatic sack tumor	**Renal and neuroblastic tumors**
Nephroblastoma (Wilms tumor)	Fetal rhabdomyoma	Cystic nephroma
Optic nerve glioma	Gastrointestinal stromal tumor	Renal angiomyolipoma
Peripheral nerve sheath tumor	Glioma of the optic pathway (with signs of NF1)	Renal cell carcinoma
Pineoblastoma	Gonadoblastoma	Renal rhabdoid tumor
Pituitary adenoma	Hemangioblastoma	**Bone and soft-tissue tumors**
Pituitary blastoma	Hepatoblastoma (*CTNNB1* wildtype)	Desmoid tumor
Renal carcinoma	Hepatocellular carcinoma	Extrarenal rhabdoid tumor
Retinoblastoma	Infantile myofibromatosis	Gardner fibroma
Rhabdomyosarcoma (not if the child is > 3 yo)	Juvenile myelomonocytic leukemia	Malignant periphery nerve sheath tumor
Spinal ependymoma	Keratocystic odontogenic tumor	Nasal chondromesenchymal hamartoma
Skin carcinoma	Large cell calcifying Sertoli-cell-tumor	**Other tumors**
Subependymal giant cell astrocytoma	Malignant peripheral nerve sheath tumor	Adrenocortical carcinoma
Thyroid carcinoma	Medullary thyroid carcinoma	Cardiac rhabdomyoma
Vestibular schwannoma	Medulloblastoma (SHH activated)	Colorectal carcinoma
Adult tumors (e.g. carcinoma, pheochromocytoma)	Medulloblastoma (WNT activated, *CTNNB1* wildtype)	Gastrointestinal stromal tumor
	Medullary renal cell carcinoma	Hepatoblastoma
	Medulloepithelioma	Medullary thyroid cancer,
	Melanoma	Ovarian Sertoli-Leydig cell tumor
	Meningioma	Parathyroid tumor
	Myelodysplastic syndrome	Pheochromocytoma,
	Myeloproliferative neoplasms (except CML)	Paraganglioma
	Myxoma	Pleuropulmonary blastoma
	Neuroendocrine tumor	Trichilemmoma
	Paraganglioma / pheochromocytoma	Small cell carcinoma of the ovary of hypercalcemic type
	Parathyroid carcinoma / adenoma	Carcinoma of the breast, lung, cervix, uterus, or bladder
	Pineoblastoma	
	Pituitary adenoma / tumor	
	Pituitary blastoma	
	Pleuropulmonary blastoma	
	Renal cell carcinoma	
	Retinoblastoma	
	Rhabdoid tumor	
	Rhabdomyosarcoma with diffuse anaplasia	
	Schwannoma	
	Schwannomatosis	
	Sertoli-Leydig cell tumor	
	Sex cord stromal tumor with annular tubules	
	Small cell carcin. of the ovary hypercalcemic type	
	Squamous cell carcinoma	
	Subependymal giant cell astrocytoma	
	Thyroid carcinoma (non-medullary)	
	Transient myeloproliferative disease	
